# Identification of novel pQTL‐SNPs associated with lung adenocarcinoma risk: A multi‐stage study

**DOI:** 10.1002/cam4.70247

**Published:** 2024-09-18

**Authors:** Yutong Wu, Huiwen Xu, Liping Mao, Rongrong Zhao, Junfeng Chu, Lili Huang, Wendi Zhang, Yiran Liu, Qiong Chen, Xiaobo Tao, Siqi Li, Shenxuan Zhou, Anhui Ning, Zhenyu Li, Tian Tian, Lei Zhang, Jiahua Cui, Guangyu Tian, Minjie Chu

**Affiliations:** ^1^ Department of Epidemiology School of Public Health, Nantong University Nantong Jiangsu China; ^2^ Department of Oncology Affiliated Nantong Hospital of Shanghai University (The Sixth People's Hospital of Nantong) Nantong Jiangsu China; ^3^ Department of Oncology Jiangdu People's Hospital of Yangzhou Yangzhou China; ^4^ Department of Critical Care Medicine Affiliated Hospital of Nantong University Nantong China; ^5^ Nantong‐Leicester Joint Institute of Kidney Science, Nephrology Affiliated Hospital of Nantong University Nantong China

**Keywords:** lung adenocarcinoma, protein quantitative trait loci (pQTL), proteomics, SNP

## Abstract

**Background and Objective:**

To explore the association between protein quantitative trait loci (pQTL‐SNPs) and the risk of LUAD.

**Methods:**

“Blood +” high depth blood proteomics analysis was performed on plasma from female LUAD patients and female healthy controls, and combined with proteomics data from tumors and adjacent non‐tumor tissues of female LUAD patients to screen proteins uniformly expressed in plasma and tissues. pQTL‐SNPs were then screened through multiple databases and subjected to multilevel screening. The associations between selected pQTL‐SNPs and LUAD risk were evaluated by Female Lung Cancer Consortium in Asia GWAS (FLCCA GWAS). Enzyme linked immunosorbent assay (ELISA) is used to determine the levels of candidate protein.

**Results:**

A total of 7 pQTL‐SNPs were significantly associated with altered LUAD risk (*p* < 0.05). Meanwhile, the expression of their corresponding target proteins were all decreased in both plasma and tumor tissues of LUAD cases, which may play a role of tumor suppressor proteins. After mutation of 3 pQTL‐SNPs (rs7683000, rs73224660, and rs2776937), the expression of corresponding target proteins BST1 and NRP1 decreased, and as potential tumor suppressor proteins, which may promote tumorigenesis and further increasing the risk of developing LUAD (OR >1, *p* < 0.05); while after mutation the other pQTL‐SNP rs62069916, the corresponding target protein APOH expression was increased, while as a potential tumor suppressor protein, which may inhibit tumorigenesis and further reduced the risk of developing LUAD (OR <1, *p* < 0.05). In addition, the expression of NRP1 and APOH were significant decreased in LUAD cell lines and validated in plasma of LUAD patients.

**Conclusion:**

A total of 4 pQTL‐SNPs (rs7683000, rs73224660, rs2776937, and rs62069916) may associate with altered LUAD risk by regulating the expression of target proteins (BST1, NRP1, and APOH) after mutation.

## 1 INTRODUCTION

Lung cancer is the leading cause of cancer death worldwide, with an estimated 2.20 million new cases and 1.79 million deaths each year.^1^, ^2^ Non‐small cell lung cancer (NSCLC) is the major pathological type of lung cancer, accounting for about 85%.^3^ Lung adenocarcinoma (LUAD), the most frequent subtype of NSCLC, causes more than 1 million deaths annually all over the world, and its five‐year survival rate is only 15%.^4^ Among men, LUAD incidence rates started to stabilize during the mid‐1980s in many countries.^5^ However, in women, the incidence rates of LUAD still present an upward trend.^6^, ^7^, ^8^ Therefore, more attention should be devoted to LUAD, especially for female patients.

The pathogenesis of LUAD is a complex process of multi‐factors, mainly resulting from the interaction between environmental and genetic factors.^9^ Studies of smokers with lung cancer showed that females have a 50% higher risk of developing LUAD than males at the same smoking levels,^10^ which may be related to greater sensitivity to tobacco carcinogenicity in females, as tobacco‐induced *TP53* mutations are more common in females.^10^ In addition to *TP53*, mutations in other carcinogenic drivers of LUAD are also more prone to occur in females, including epidermal growth factor receptor, kirsten rat sarcoma viral oncogene homolog, and anaplastic lymphoma receptor tyrosine kinase.^11^ Therefore, acquiring more characteristic genetic factors related to the risk of female LUAD might provide new strategies for diagnostics and therapy of female LUAD.

Although genome‐wide association studies (GWASs) have identified many single nucleotide polymorphisms (SNPs) associated with LUAD risk, most of them are located in non‐coding regions.^12^, ^13^ It is still challenging to elucidate the molecular mechanisms underlying these genetic variants. Quantitative trait loci (QTL) research may help researchers to explore the related potential mechanisms. Currently, many studies focus on the expression quantitative trait locus (eQTL), which studies SNPs related to the transcriptional abundance of mRNA or other transcripts. However, mRNA expression correlates poorly with protein levels of many genes, partly because of many post‐transcriptional regulations,^14^ and eQTLs do not yet explain changes in protein levels well. Proteins are the end products of post‐translationally modified and processed transcripts and contain further information that is not detectable at the transcriptome level.^15^ Thus, in‐depth studies of proteins might help explain the causal relationship between genetic variations and diseases, including LUAD.

Genetic variants can influence protein abundance in a quantitative manner, and protein quantitative trait loci (pQTL) analysis can help identify genetic variants that influence protein abundance.^16^ Thus, exploring the relationships among pQTL‐SNPs, LUAD‐related proteins, and LUAD susceptibility may provide new diagnostic and therapeutic avenues for LUAD. Nevertheless, population‐based studies with large samples focusing on pQTL‐SNPs and the risk of LUAD are still lacking. This study intended to identify pQTL‐SNPs associated with female LUAD risk.

## 2 MATERIALS AND METHODS

### 2.1 Study population

In the “Blood+” high‐depth blood proteomic screening analysis, 10 female LUAD cases and 10 age‐matched healthy female controls were recruited from Hai'an People's Hospital, Jiangsu Province, China, between August 2021 and Novomber 2021.

In the validation phase, we recruited additional 50 female LUAD patients from Hai'an People's Hospital from December 2021 to 2022 and 100 age matched female healthy controls in a ratio of 1:2 case population and healthy controls. The healthy controls were subjects who participated in health examinations from Jiangdu District, Yangzhou City, Jiangsu Province from August 2022 to September 2022. All LUAD patients were confirmed by pathology. All subjects obtained written informed consent and were reviewed and approved by the Ethics Committee of Nantong University (Approval No.2022‐2). All female samples were from non‐smokers.

### 2.2 Proteomics analysis in plasma

**FIGURE 1:**
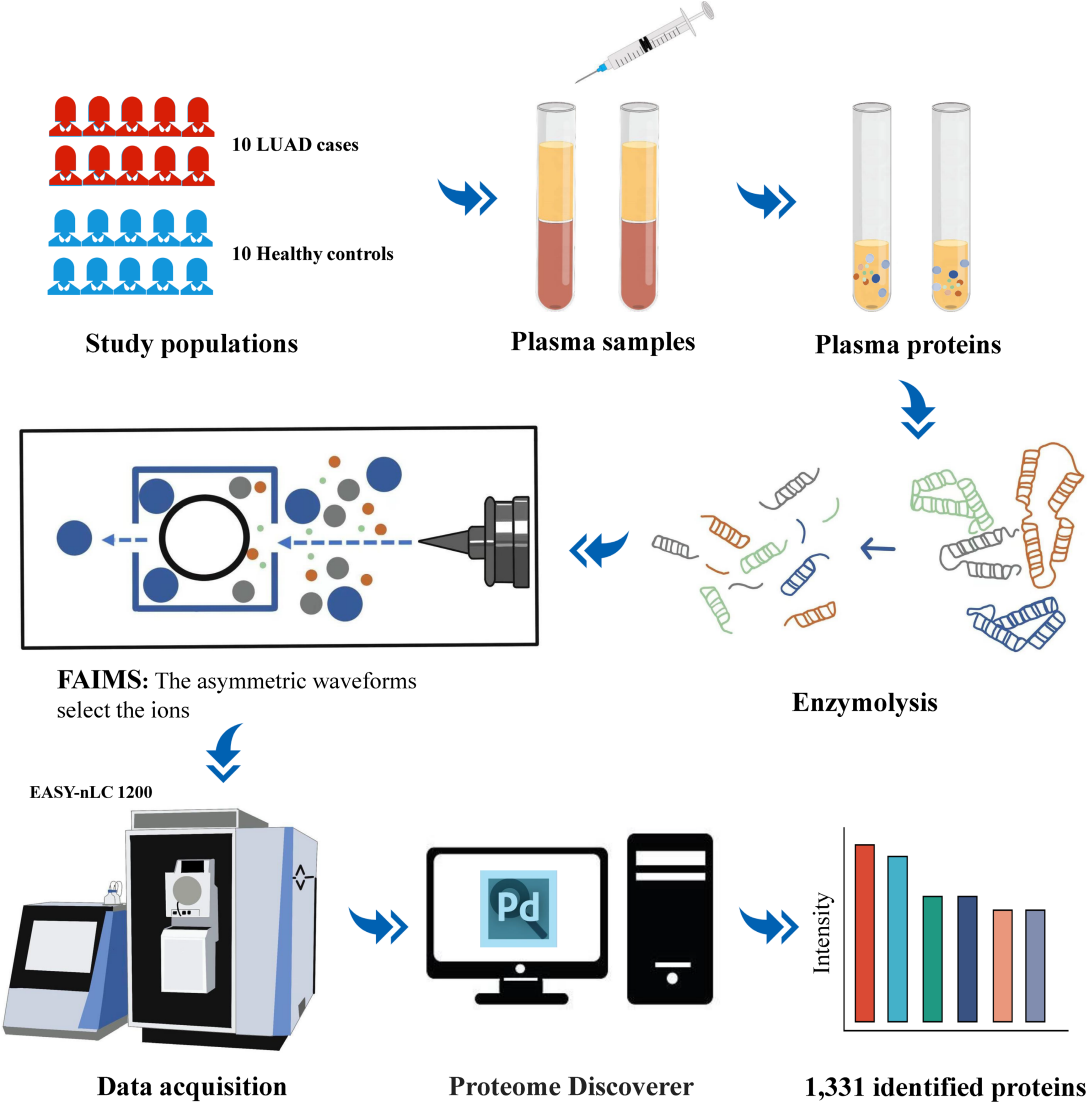
“Blood+” high depth blood proteomics analysis. FAIMS, high‐field asymmetric waveform ion mobility spectrometry; LUAD, lung adenocarcinoma.

Proteomics analysis in plasma was conducted by Jingjie PTM BioLabs (Hangzhou, China). Primary experimental procedures for “Blood+” high‐depth blood proteomics analysis included protein extraction, trypsin digestion, liquid chromatography–tandem mass spectrometry (LC–MS/MS) analysis based on high‐field asymmetric waveform ion mobility spectrometry (FAIMS) technology. The resulting MS/MS data were processed using Proteome Discoverer (v2.4.1.15) search engine (v.1.6.15.0). database Homo_sapiens_9606_PR_20210721.fasta (78120 sequences) with a decoy for FDR calculation; Trypsin (Full) enzyme, 2 missed cleavages; minimum peptide length 6 amino acids, maximum 3 modifications per peptide; precursor ion mass tolerance 10 ppm, fragment ion mass tolerance 0.02 Da; Set Carbamidomethyl (C) to a fixed modification, and set Oxidation (M), Acetyl (N‐terminus), Met‐loss (M), Met‐loss+acetyl (M) to a variable modification. FDR was adjusted to <1% (Figure 1).

### 2.3 Data sources of proteomics analysis in tissues

Proteins with differential expression between 65 paired female LUAD tumor tissues and adjacent non‐tumor tissues are derived from the released proteomics data in the comprehensive proteomics study of LUAD in the Chinese population.^17^ The study, published in *Cell* in 2020 by Xu et al.,^17^ successfully constructed a proteomics‐based molecular panorama of LUAD by conducting a comprehensive proteomic analysis of 103 Chinese LUAD patients and integrating genomic signature data and clinical information. In this study, 103 primary LUAD tumor tissues with paired adjacent non‐tumor tissues were collected from untreated Chinese patients for a comprehensive proteomic analysis. Among them, the LUAD samples contained 65 female patients and 38 male patients. Of these, protein data from 65 paired female LUAD tumor tissues and adjacent non‐tumor tissues were used in this study.

### 2.4 Selection of LUAD‐related proteins that were consistently differentially expressed in both plasma and tissues

**FIGURE 2:**
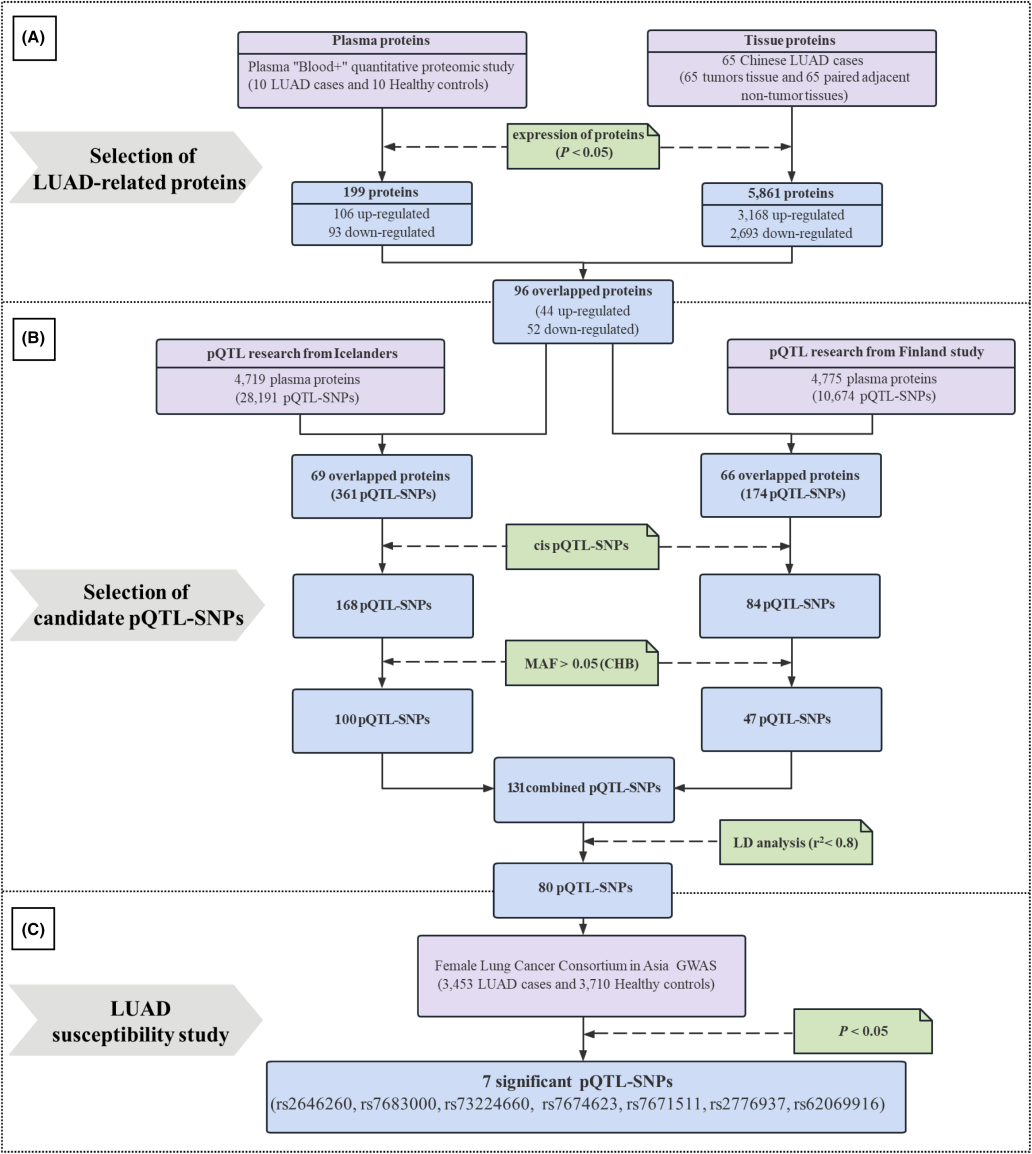
Schematic representation of the study design. CHB, Chinese Han population in Beijing; LD, linkage disequilibrium; LUAD, lung adenocarcinoma; MAF, minor allele frequency; pQTL, protein quantitative trait loci; SNPs, single nucleotide polymorphisms.

Based on the plasma from the 10 female LUAD cases and 10 healthy controls, we performed the “Blood+” high‐depth blood proteomic analysis to screen the differential proteins with *p* < 0.05. Meanwhile, according to the proteomic data of tumor tissues and adjacent non‐tumor tissues from female LUAD cases from Chinese female patients,^17^ the differential expressed proteins were selected (*p* < 0.05). Then, the overlapped proteins, consistently differential expressed in both plasma and tumor tissues were obtained (Figure 2A).

### 2.5 Selection of candidate pQTL‐SNPs in LUAD‐related proteins

Two pQTL‐SNPs databases were used to help identify pQTL‐SNPs associated with the above‐obtained proteins.^18^, ^19^ One database conducted by Ferkingstad et al. found 28,191 pQTL‐SNPs from 4719 proteins and 27.2 million variants in 35,559 Icelanders.^18^ The other database was conducted by *Pietzner* et al., and it revealed 10,674 pQTL‐SNPs from 4775 proteins and 10.2 million genetic variants in 10,708 Finnish participants.^19^ According to the above‐screened LUAD‐related proteins, we selected corresponding cis pQTL‐SNPs from the two pQTL‐SNPs databases respectively. Then, for each database, pQTL‐SNPs with minor allele frequency (MAF) >0.05 in the Chinese Han population were selected. Furthermore, the combined pQTL‐SNPs were collected. The final pQTL‐SNPs were determined from the combined ones by linkage disequilibrium (LD) analysis with an *r* ^2^ threshold of 0.80 (Figure 2B).

### 2.6 Association analysis between candidate pQTL‐SNPs and the risk of female LUAD

We performed the association analysis between candidate pQTL‐SNPs and female LUAD risk on the basis of the Female Lung Cancer Consortium in Asia (FLCCA) GWAS. The FLCCA GWAS was from the database of Genotypes and Phenotypes (dbGAP), with accession number phs000716.v1.p1. This GWAS included 3453 female LUAD patients who never smoked and 3710 healthy controls from 14 studies in China, Korea, Japan, and Singapore^20^ (Figure 2C).

### 2.7 Cell line selection and culture

The LUAD cell lines used in this study were PC9, NCI‐A549, and SPC‐A‐1, as well as HBE (human bronchial epithelial cell line), all cell lines purchased from American Type Culture Collection (ATCC). All cell lines were preserved in DMEM medium supplemented with 10% fetal bovine serum, 100 U/mL penicillin, and 100% μ G/mL streptomycin cultured in an incubator containing 5% CO2 at 37°C.

### 2.8 Western blot

Western blot analysis was conducted as previously described.^21^ The antibodies we used were anit‐BST1 (1:500, Proteintech, China), anti‐NRP1 (1:1000, Proteintech, China), anti‐APOH (1:2000, Proteintech, China). The protein level of BST1, NRP1 and APOH was standardized by anti‐GAPDH (1:1000, Beyotime, China). Image J software (Version 1.54) was used for gray scale analysis of protein bands. Western blot analysis for each protein cell line was independently repeated 3 times.

### 2.9 Enzyme linked immunosorbent assay

In the validation phase, enzyme linked immunosorbent assay (ELISA) is used to determine the levels of BST1, NRP1, and APOH proteins in plasma samples from 50 LUAD cases and 100 healthy controls. The collected plasma was centrifuged at 2000 rpm and sampled, stored at 4°C for 20 min, and stored at −80°C until analysis. Then, the protein levels in human plasma samples were measured using the double antibody sandwich method using BST1, NRP1, and APOH kits (ELISA kits purchased from Enzyme Immunoassay Biotechnology Co., Ltd. in Yancheng, Jiangsu, China). Finally, the absorbance of each well at 450 nm was measured using enzyme‐linked immunosorbent assay (Labsystems Multikan) to calculate protein expression.

### 2.10 Statistical analysis

The data on protein expression from both plasma and tissues were processed using the same normalization method in this study. Differences in protein expression levels between LUAD cell lines (PC9, NCI‐A549, and SPC‐A‐1) and HBE (human bronchial epithelial cell line), as well as differences in the plasma validation phase between LUAD patients and healthy controls were analyzed using the Student's *t*‐tests. Regression coefficient (*β*) was used to assess the association between the different genotypes of candidate pQTL‐SNPs and target protein expression. Logistic regression analysis was used to estimate the association between the candidate pQTL‐SNPs and LUAD risk, and the age‐adjusted odds ratios (ORs) and 95% confidence intervals (95% CIs) were obtained. *p* < 0.05 was considered statistically significant. All statistical analyses were performed using R 4.1.1.

## 3 RESULTS

### 3.1 Characteristics of the Study Subjects

In this study, the baseline characteristics of the study subjects are shown in Table 1. Briefly, 10 female LUAD patients and 10 healthy female controls were enrolled in screening differentially expressed plasma proteins. And 65 paired female LUAD tumor tissues and adjacent non‐tumor tissues were used to screen differentially expressed proteins in tissues. In the differential expression study of plasma proteins, the age distribution of controls was consistent with that of the case group. In the differential expression study of tissue proteins, half of the patients were over 60 years old. In the FLCCA GWAS, 51.67% of cases were over 60 years old, while 45.74% of controls were over 60 years old. During the plasma validation phase, there was also no statistical difference in age distribution between LUAD patients and healthy female controls (Table 1).

**TABLE 1:** Characteristics of the subjects enrolled in this study.

Variables	Plasma (Blood+)			Plasma (validation)			Tissue	FLCCA	
	Case (*n* = 10)	Control (*n* = 10)	*p*‐value	Case (*n* = 50)	Control (*n* = 100)	*p*‐value	Case (*n* = 65)	Case (*n* = 3453)	Control (*n* = 3710)
Age, *n* (100%)									
≤60	3 (30)	3 (30)	1.000	27 (54)	46 (46)	0.390	32 (49.23)	1669 (48.33)	2013 (54.26)
>60	7 (70)	7 (70)		23 (46)	54 (54)		33 (50.77)	1784 (51.67)	1697 (45.74)
Stage, *n* (100%)									
I	8 (80)			46 (92)			33 (50.77)		
II	0 (0)			4 (8)			10 (15.38)		
III	2 (20)			0 (0)			21 (32.31)		
IV	0 (0)			0 (0)			1 (1.54)		

### 3.2 Identification of LUAD‐related proteins that were consistently differentially expressed in both plasma and tissues

The “Blood+” high‐depth blood proteomic analysis on the plasma identified 1331 proteins. Among these proteins, 199 differential expressed proteins were selected, of which 106 were up‐regulated and 93 were down‐regulated in the plasma of female LUAD (*p* < 0.05). Meanwhile, based on 65 paired tumor tissues and adjacent non‐tumor tissues of female LUAD, 5861 differential expressed proteins were collected. Taking the intersection of the selected differentially expressed proteins from plasma and tissues, 96 LUAD‐related proteins were determined, of which 44 were up‐regulated and 52 were down‐regulated (Figure 2A).

### 3.3 Selection of candidate pQTL‐SNPs in LUAD‐related proteins

We used two publicly available pQTL‐SNPs databases to obtain pQTL‐SNPs on these 96 LUAD‐related proteins. The pQTL‐SNPs research from Iceland contained 28,191 pQTL‐SNPs acting on 4719 plasma proteins. Based on the intersection of the 4719 proteins and the previously identified 96 LUAD‐related proteins, 69 proteins and 361 related pQTL‐SNPs were identified, including 168 cis pQTL‐SNPs. Then, 100 pQTL‐SNPs were obtained by screening for MAF >0.05 in the Chinese Han population. The other pQTL‐SNPs research from Finland contained 10,674 pQTL‐SNPs acting on 4775 plasma proteins. Similar to the screening process of the above pQTL‐SNPs research, 47 pQTL‐SNPs with MAF >0.05 in the Chinese Han population were collected. Subsequently, we merged the two datasets of pQTL‐SNPs into 131 combined pQTL‐SNPs. Next, the combined pQTL‐SNPs were further screened by LD analysis with an *r* ^2^ threshold of 0.80, and 80 pQTL‐SNPs were obtained **(**Figure 2B).

### 3.4 Association between 80 candidate pQTL‐SNPs and female LUAD risk in FLCCA GWAS

The association between 80 candidate pQTL‐SNPs and female LUAD risk was evaluated in the FLCCA GWAS database (Table S1). And the results showed that 7 pQTL‐SNPs were significantly associated with altered LUAD risk in females (*p* < 0.05) (Table S1, Figure 2C). Among which, the variant alleles of 5 pQTL‐SNPs (rs7683000, rs73224660, rs7674623, rs7671511 and rs2776937) were significantly associated with increased LUAD risk in females (additive model: OR >1, *p* < 0.05). Meanwhile, the variant alleles of the other 2 pQTL‐SNPs (rs2646260 and rs62069916) were significantly associated with decreased LUAD risk in females (additive model: OR <1, *p* < 0.05) (Table 2).

**TABLE 2:** The association between identified 7 pQTL‐SNPs and LUAD risk in females.

SNPs^a^	Gene	Location (hg37)	Genotypes	Cases, *n* (100%)	Controls, *n* (100%)	Adjusted OR (95% CI)^a^	*p*‐Value^a^
rs2646260	*COL6A3*	chr2:238277795	AA	1802 (52.19)	1829 (49.30)	1 (ref)	‐
			AG	1366 (39.56)	1527 (41.16)	0.91 (0.82–1.00)	0.057
			GG	285 (8.25)	354 (9.54)	0.82 (0.69–0.97)	0.022
			Dominant model			0.89 (0.81–0.98)	0.016
			Recessive model			0.86 (0.73–1.01)	0.061
			Additive model			0.91 (0.84–0.97)	0.008
rs7683000	*BST1*	chr4:15647074	AA	2027 (58.70)	2264 (61.02)	1 (ref)	‐
			AG	1224 (35.45)	1252 (33.75)	1.09 (0.99–1.21)	0.079
			GG	202 (5.85)	194 (5.23)	1.16 (0.94–1.42)	0.165
			Dominant model			1.10 (1.00–1.21)	0.045
			Recessive model			1.12 (0.91–1.37)	0.272
			Additive model			1.08 (1.00–1.17)	0.040
rs73224660	*BST1*	chr4:15714762	GG	2067 (59.86)	2330 (62.80)	1 (ref)	‐
			GA	1210 (35.04)	1208 (32.56)	1.13 (1.02–1.25)	0.018
			AA	176 (5.10)	172 (4.64)	1.15 (0.93–1.43)	0.206
			Dominant model			1.13 (1.03–1.24)	0.012
			Recessive model			1.10 (0.89–1.37)	0.370
			Additive model			1.10 (1.02–1.19)	0.014
rs7674623	*ANTXR2*	chr4:80794681	CC	3131 (90.67)	3411 (91.94)	1 (ref)	‐
			CT	311 (9.01)	293 (7.90)	1.16 (0.98–1.37)	0.085
			TT	11 (0.32)	6 (0.16)	2.04 (0.75–5.53)	0.162
			Dominant model			1.18 (1.00–1.39)	0.054
			Recessive model			2.01 (0.74–5.46)	0.169
			Additive model			1.18 (1.01–1.39)	0.037
rs7671511	*SPARCL1*	chr4:88449181	CC	1723 (49.90)	1897 (51.13)	1 (ref)	‐
			CT	1405 (40.69)	1525 (41.11)	1.02 (0.92–1.12)	0.744
			TT	325 (9.41)	288 (7.76)	1.25 (1.05–1.49)	0.011
			Dominant model			1.05 (0.96–1.16)	0.271
			Recessive model			1.24 (1.05–1.47)	0.011
			Additive model			1.07 (1.00–1.16)	0.049
rs2776937	*NRP1*	chr10:33606189	GG	1213 (35.13)	1361 (36.68)	1 (ref)	‐
			GA	1670 (48.36)	1794 (48.36)	1.05 (0.95–1.16)	0.350
			AA	570 (16.51)	555 (14.96)	1.16 (1.00–1.33)	0.044
			Dominant model			1.07 (0.98–1.18)	0.144
			Recessive model			1.12 (0.99–1.28)	0.074
			Additive model			1.07 (1.00–1.14)	0.049
rs62069916	*APOH*	chr17:64280503	CC	1224 (35.45)	1268 (34.18)	1 (ref)	‐
			CT	1642 (47.55)	1736 (46.79)	0.98 (0.89–1.09)	0.729
			TT	587 (17.00)	706 (19.03)	0.86 (0.75–0.98)	0.028
			Dominant model			0.95 (0.86–1.04)	0.268
			Recessive model			0.87 (0.77–0.98)	0.023
			Additive model			0.94 (0.88–1.00)	0.047

^a^ Logistic regression analysis adjusted for age.

### 3.5 Expression of the acting proteins of the 7 identified pQTL‐SNPs in plasma and tissues

**FIGURE 3:**
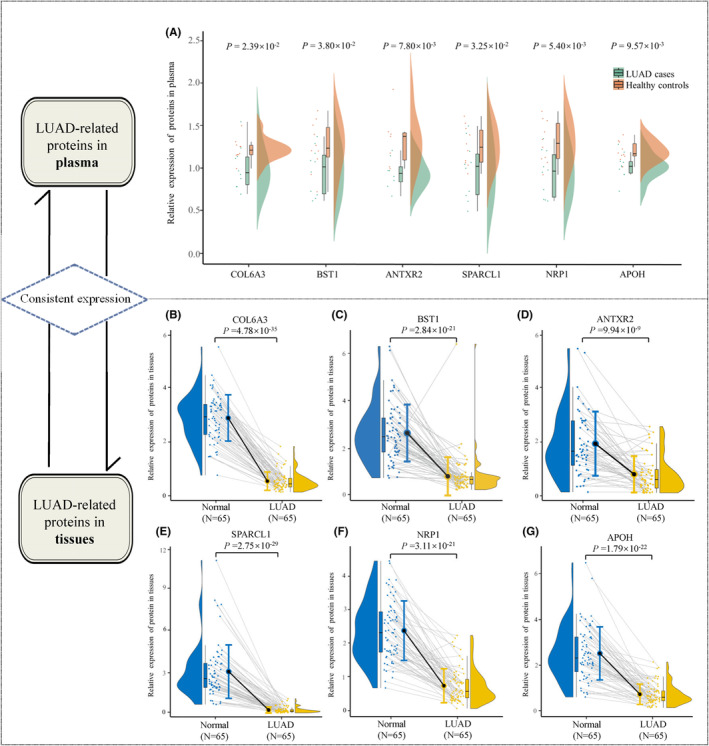
Relative expression of LUAD‐related proteins in plasma and tissues. (A) Relative expression of LUAD‐related proteins (COL6A3, BST1, ANTXR2, SPARCL1, NRP1, and APOH) in plasma. (B) Relative expression of COL6A3 in tissues. (C) Relative expression of BST1 in tissues. (D) Relative expression of ANTXR2 in tissues. (E) Relative expression of SPARCL1 in tissues. (F) Relative expression of NRP1 in tissues. (G) Relative expression of APOH in tissues.

Among the 7 identified pQTL‐SNPs, two of which (rs7683000 and rs73224660) were located on BST1, while the other five (rs2646260, rs7674623, rs7671511, rs2776937 and rs62069916) were located on COL6A3, ANTXR2, SPARCL1, NRP1, and APOH, respectively. Proteomics analysis in plasma showed that the protein expression levels of the six proteins in plasma in female LUAD cases were both significantly lower than those in healthy controls (*p* < 0.05) (Figure 3A). Consistently, compared with adjacent non‐tumor tissues, the expression levels of the six proteins were both significantly lower in tumor tissues (*p* < 0.05) (Figure 3B–G). The results of protein expression analysis showed that the six proteins could play roles as tumor suppressor proteins both in plasma and tissues.

### 3.6 Relationship between genotypes and protein expression of the identified 7 pQTL‐SNPs

**FIGURE 4:**
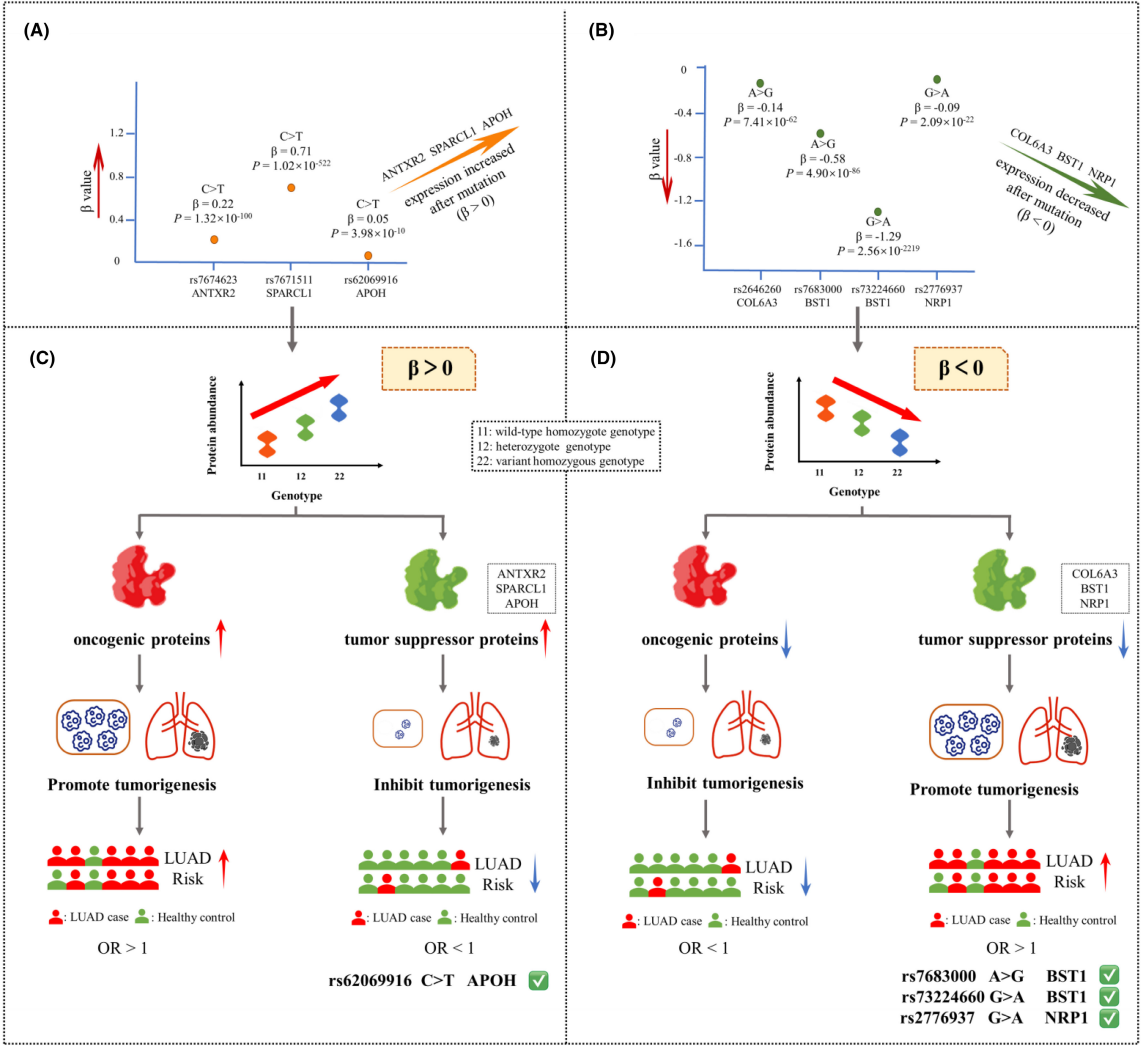
The relationship between the 7 pQTL‐SNPs and their target protein expression and the diagram of pQTL‐SNPs mediating changes in LUAD risk after mutation. (A) The expressions of target proteins were higher under the variant alleles of corresponding pQTL‐SNPs. (B) The expressions of target proteins were lower under the variant alleles of corresponding pQTL‐SNPs. (C) Mutation of pQTL‐SNPs leads to increased protein expression which in turn mediates changes in LUAD risk. (D) Mutation of pQTL‐SNPs leads to reduced protein expression which in turn mediates changes in LUAD risk.

To further investigate the relationship between genotype and protein expression for the above 7 pQTL‐SNPs, we obtained the corresponding β and *P* values in both databases as a way to demonstrate the relationship between genotype and protein expression. Among them, the rs7674623 located on *ANTXR2* (*p* = 1.32 × 10^−100^), the rs7671511 located on *SPARCL1* (*p =* 1.02 × 10^−522^), and the rs62069916 located on *APOH* (*p =* 3.98 × 10^−10^) had significantly higher target protein expression under the variant alleles (*β* > 0) (Figure 4A), while the rs2646260 located on *COL6A3* (*p* = 7.41 × 10^−62^), the rs7683000 located on *BST1* (*p* = 4.90 × 10^−86^), the rs73224660 located on *BST1* (*p* = 2.56 × 10^−2219^), and the rs2776937 located on *NRP1* (*p* = 2.09 × 10^−22^) had significantly lower target protein expression under the variant alleles (*β* < 0) (Figure 4B).

### 3.7 Regulation patterns among pQTL‐SNP, protein expression and LUAD risk in females

As shown in Figure 4C, *β* > 0 indicates that the expression of the target protein increased after mutation of the corresponding pQTL‐SNPs, it is biologically plausible that if the expressions of oncogenic proteins were elevated after pQTL‐SNP mutation, then subjects may have an increased risk of developing LUAD (OR >1); on the contrary, it is also biologically plausible that if the expression of tumor suppressor protein is elevated after pQTL‐SNPs mutation, then subjects may have an decreased risk of developing LUAD (OR <1). Similarly, *β* < 0 indicates that the expression of the target protein decreased after mutation of the corresponding pQTL‐SNPs, it is biologically plausible that if the expressions of oncogenic proteins were reduced after pQTL‐SNPs mutation, then subjects may have an decreased risk of developing LUAD (OR <1); on the contrary, it is also biologically plausible that if the expression of tumor suppressor protein is reduced after pQTL‐SNP mutation, then subjects may have an increased risk of developing LUAD (OR >1) (Figure 4D).

Considering the six proteins mentioned above could all play roles as tumor suppressor proteins both in plasma and tissues. In this regard, there are 4 pQTL‐SNPs meet the biologically plausible regulation patterns. Specifically, after mutation of the other pQTL‐SNP (rs62069916 C > T), the corresponding target protein APOH expression was increased, while as a tumor suppressor protein, increased expression of which may inhibit tumorigenesis and further reduced the risk of developing LUAD (OR <1, *p* < 0.05) (Figure 4C, right); while after mutation of 3 pQTL‐SNPs (rs7683000 A > G, rs73224660 G > A, and rs2776937 G > A), the expression of corresponding target proteins BST1and NRP1 decreased, and as tumor suppressor proteins, decreased expression of which may promote tumorigenesis and further increasing the risk of developing LUAD (OR >1, *p* < 0.05) (Figure 4D, right).

### 3.8 Protein expression of BST1, NRP1, and APOH in LUAD cell lines

**FIGURE 5:**
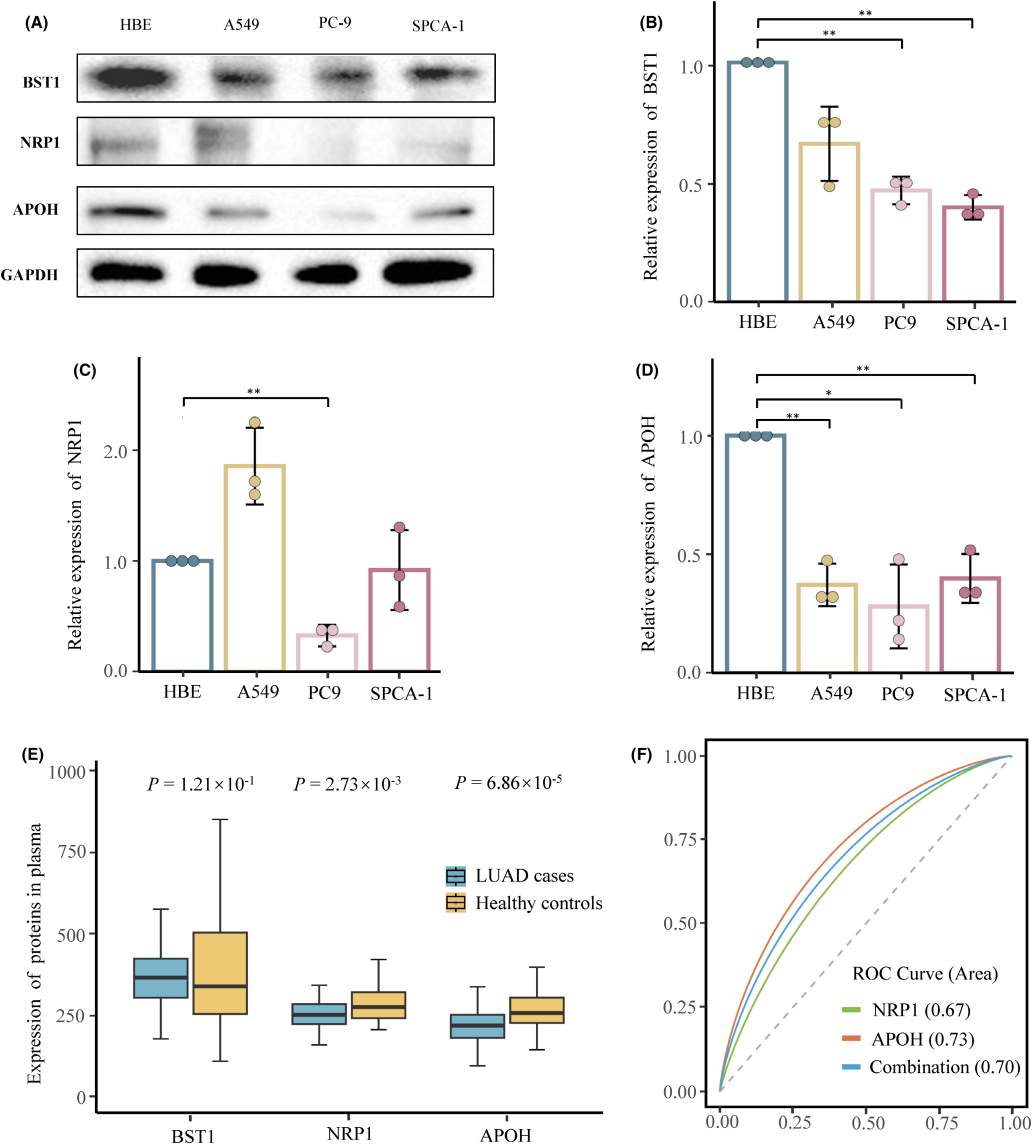
Validation of BST1, NRP1 and APOH proteins. (A–D) Comparison of the expression of BST1, NRP1 and APOH in LUAD cell lines and normal cell lines by western blot. The expression GAPDH is used as a reference. Each experiment was independently repeated three times and normalized. (B) BST1, (C) NRP1 and (D) APOH were quantified for relative expression levels. *p < 0.05, **p < 0.01. (E, F) (E) Comparison of plasma BST1, NRP1 and APOH protein levels between female LUAD patients and female healthy controls by ELISA. (F) ROC curves of NRP1, APOH and their combined levels in LUAD patients and healthy controls. ELISA, enzyme‐linked immunosorbent assay; ROC, receiver operating characteristic.

To determine the expression of BST1, NRP1, and APOH in LUAD cell lines, we performed protein blotting on LUAD cell lines and normal bronchial epithelial (HBE) cell line (Figure 5A). The results showed that the expressions of BST1 in PC9 and SPCA‐1 were significantly lower than that in HBE (*p* < 0.01). The expression of NRP1 in PC9 was significantly lower than that in HBE (*p* < 0.01). APOH expression levels were lower in A549 (*p* < 0.01), PC9 (*p* < 0.05) and SPCA‐1 (*p* < 0.05) than those in HBE (Figure 5B–D).

### 3.9 Downregulation of plasma NRP1 and APOH protein expression in female LUAD patients

ELISA results showed that the plasma protein expressions of NRP1 (*p* = 2.73 × 10^−3^) and APOH (*p* = 6.86× 10^−5^) were significantly lower than that in the healthy female control group (Figure 5E).

We then used the plasma protein levels of the case (*n* = 50) and control group (*n* = 100) from the validation phase to calculate the receiver operating characteristic (ROC) curves and the area under the ROC curves (AUC): the ROC curve showed APOH (AUC = 0.73, 95% CI: 0.57–0.75, *p* < 0.001) may be used as an indicator to distinguish cases from controls (Figure 5F).

## 4 DISCUSSION

In a case–control study based on FLCCA GWAS of 3453 female LUAD cases and 3710 healthy controls, we identified 7 pQTL‐SNPs were significantly associated with the altered risk of developing LUAD. The expressions of the corresponding proteins were both decreased in plasma and tumor tissues of LUAD cases, playing the role of tumor suppressor proteins. Among them, it is biologically plausible that 4 pQTL‐SNPs (rs7683000, rs73224660, rs2776937, and rs62069916) may be associated with LUAD risk by regulating the expression of target proteins (BST1, NRP1, and APOH) after mutation.

The rs7683000 and rs73224660 are located in the intron region of *BST1*, rs2776937 is located in the intron region of *NRP1*, and rs62069916 is located in the intron region of *APOH*. The three proteins (BST1, NRP1, and APOH) may function as tumor suppressor proteins in LUAD. BST1 is a cell adhesion glycoprotein encoded by the human bone marrow stromal cell 1 (*BST1*) gene. Initially found in bone marrow stromal cells, it is essential for the growth and development of B lymphocytes.^22^ BST1 is a multifunctional molecule. It is a metabolic enzyme that catalyzes the synthesis of cyclic Adp‐beta‐d‐ribose (cADPR) from NAD and hydrolyzes it to ADp‐d‐ribose (ADPR) and plays an essential role in Ca^2+^ homeostasis.^23^ BST1 is also an immunoregulatory molecule and is widely present in immune cells. Besides, BST1 may play crucial roles in mediating white blood cell migration, etc. Studies have shown that BST1 glycoprotein is overexpressed in malignant pleural mesothelioma, a marker of enhanced tumor aggressiveness, and contributes to diagnosing malignant mesothelioma.^24^ And BST1 overexpression promotes the progression of epithelial ovarian cancer by promoting mesenchymal differentiation^25^ and is an independent prognostic factor for overall survival.^26^ In addition, BST1 protein is associated with, or implicated in, pulmonary fibrosis.^27^

NRP1 (Neuropilin‐1) is a single‐pass transmembrane glycoprotein and one of the two homologous subtypes of neuroprotein (*NRP*).^28^ NRP1 has a variety of biologically functions by binding to multiple ligands. NRP1 is known to bind to class 3 semaphorins (SEMA3) coreceptor for vascular endothelial growth factor (VEGF), VEGF‐C/D, and to assist in transforming growth factor‐β (TGF‐β) signaling pathway.^29^ A large number of studies have shown that NRP1 plays a key role in tumorigenesis and progression, being involved in angiogenesis, cancer migration and tumor immunity,^30^, ^31^ and overexpression has been detected in a variety of metastatic tumors, such as breast cancer^32^ and gastric cancer.^33^ However, the role of NRP1 in some tumors may be different; for example, the high expression of NRP1 in human pancreatic cancer cells Panc‐1 can reduce its incidence.^30^ In the lung, the TGF‐β signaling pathway is associated with the development of lung tumors, and the up‐regulation of TGF‐β ligand has been observed in major lung diseases.^34^ NRP1 can promote TGF‐β1‐induced epithelial mesenchymal transition (EMT), but TGF‐β signaling pathway has a feedback effect on NRP1, which may inhibit the expression of NRP1, leading to the decline of NRP1 expression.^35^

APOH, also known as β‐2‐glycoprotein 1, belongs to the Apolipoprotein family and is a unique five‐domain plasma glycoprotein. It has three disulfide bonds in the C‐terminal V domain and a lysine‐rich positively charged region at the C‐terminal extension,^36^ thus binding to various negatively charged substances such as heparin, phospholipids, and glucan sulfates. APOH is the crucial antigen in the autoimmune disease antiphospholipid syndrome (APS).^37^ APOH is an important antithrombotic protein with potential advantages in anti‐angiogenesis processes. In addition, circulating levels of APOH produce antiangiogenic effects that inhibit tumor growth in melanoma, bladder cancer, and prostate cancer.^38^ Borlak et.al found that APOH was significantly downregulated in lung atypical adenomatous hyperplasia (AAH).^39^

A combination of eQTL and pQTL analysis might bridge the gap between SNP variants and protein level alterations, explaining the molecular mechanism of genetic variation more comprehensively. Among the 4 pQTL‐SNPs (rs7683000, rs73224660, rs2776937, and rs62069916) mentioned above, evidence from VannoPortal (http://www.mulinlab.org/vportal /index.html) supports that rs7683000, rs73224660, and rs62069916 are associated with gene expression in many human tissues (Table 3). More specifically, we performed eQTL analysis of the above 4 pQTL‐SNPs in lung tissues from the GTEx database (https://gtexportal.org/home/), which revealed that different genotypes of rs7683000 and rs73224660 located on *BST1* were both significantly associated with *BST1* expression in lung tissue samples (*p* = 1.9 × 10^−2^, *p* = 4.1 × 10^−2^). And *BST1* expression decreased after mutation in eQTL analysis, which was consistently in the same direction as the pQTL analysis. The eQTL‐SNPs rs7683000 A > G, and rs73224660 G > A caused decreased gene expression in *BST1*, which may result in decreased BST1 protein expression after translation; and as pQTL‐SNPs, BST1 protein expression was reduced after mutation, which together with eQTL may further promotes tumorigenesis leading to LUAD risk changes.

**TABLE 3:** Functional annotation for the 4 pQTL‐SNPs.

SNP	Protein	eQTLin tissues	eQTL in lung (GTEx)	Motifs altered	TF binding evidence	TF sites	Genome‐scale pathogenicity score	Genome‐scale oncogenicity score
rs7683000	BST1	21 tissues	0.019	12 changed	1	ZFX	Likely pathogenic	Likely cancer driver
rs73224660	BST1	17 tissues	0.041	14 changed			Likely pathogenic	
rs2776937	NRP1		0.77	10 changed	4	ZNF600, RXRA, ZNF140, EP300	Likely pathogenic	Likely cancer driver
rs62069916	APOH	4 tissues	0.70	28 changed			Likely pathogenic	Likely cancer driver

Abbreviations: eQTL, expression quantitative trait locus; SNP, single nucleotide polymorphism; TF, transcript factor.

**FIGURE 6:**
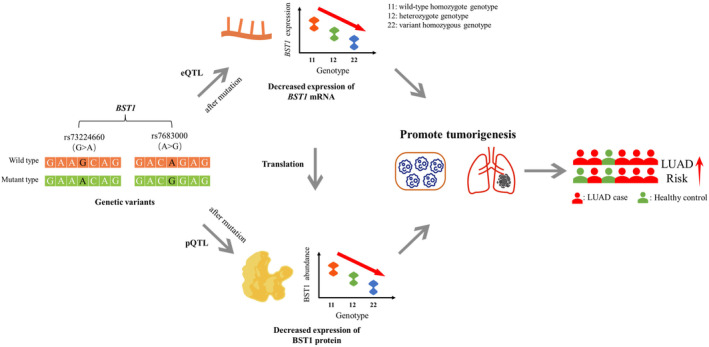
Network of mutual regulation of pQTL, eQTL and LUAD risk. eQTL, expression quantitative trait loci; pQTL, protein quantitative trait loci.

Besides, functional annotation in VannoPortal suggests that all 4 pQTL‐SNPs mutant genotypes may potentially increase motif factor binding affinity (Table 3). Among them, rs7683000 may affect the transcript factor (TF) binding of ZFX; rs2776937 may affect the TF binding of ZNF600, RXRA, ZNF140, and EP300. In terms of variant pathogenicity, the 4 pQTL‐SNPs are possible pathogenic mutations supported by genome‐scale base‐wise pathogenicity prediction methods (fitCons). In addition, rs7683000, rs2776937, and rs62069916 are possible cancer driver mutations supported by FunSeq2 or regBase‐CAN tools. The predicted correlation information may be useful for explaining how mutations in these 4 pQTL‐SNPs lead to abnormal activities of certain transcriptional or post‐transcriptional factors that regulate the expression of target proteins, which may be expected to provide new directions for resolving the regulatory role of important functional pQTL‐SNPs (Figure 6).

The present study has the following focus. First, we comprehensively applied the multi‐omics data, and obtained consistently differentially expressed LUAD‐related proteins in both tissues and plasma, which may effectively improve the reliability of population susceptibility studies. Meanwhile, we precisely obtained pQTL‐SNPs associated with identified female LUAD‐related proteins through two published convincing databases (the database from plasma protein level test in Icelandic population is published in Nature Genetics in 2021; the database from plasma protein level test in Finnish population is published in Science in 2021), which may increase the possibility of obtaining pQTL‐SNPs related to female LUAD susceptibility. Finally, this study used a large FLCCA GWAS database (3453 LUAD cases and 3710 healthy controls), which may make the results of population susceptibility study more reliable and convincing. The discovered genetic susceptibility markers may also expect to help predict the risk of individual LUAD risk, especially in females.

Although our article identified seven pQTL‐SNPs that may influence the risk of LUAD in female, there are still some limitations. The database we used for identifying pQTL‐SNPs is sourced from Icelanders and Finland, which does not include the Chinese population. Even though we validated candidate pQTL‐SNPs in Asian populations in subsequent studies, ethnic differences might cause biases in the results for the Chinese population. Therefore, future research will further explore the genetic characteristics of the Chinese population and compare them with other ethnicities. This comprehensive approach aims to deepen our understanding of how genetic variations manifest across different populations, thereby enhancing the reliability and applicability of research findings.

## 5 CONCLUSION

In conclusion, we discovered that genetic mutations in 7 pQTL‐SNPs may affect the risk of developing LUAD in females. Among them, 4 pQTL‐SNPs (rs7683000, rs73224660, rs2776937, and rs62069916) may associate with altered LUAD risk by regulating the expression of target proteins (BST1, NRP1, and APOH) after mutation. Our findings may provide clues to understand and explore how genetic variants contribute to protein levels and help investigate the mechanisms of LUAD development.

## AUTHOR CONTRIBUTIONS

**Yutong Wu:** Conceptualization (equal); writing – original draft (equal); writing – review and editing (equal). **Huiwen Xu:** Conceptualization (equal); formal analysis (equal); writing – original draft (equal); writing – review and editing (equal). **Liping Mao:** Data curation (equal); writing – review and editing (equal). **Rongrong Zhao:** Formal analysis (equal); writing – review and editing (equal). **Junfeng Chu:** Formal analysis (equal); writing – review and editing (equal). **Lili Huang:** Formal analysis (equal); writing – review and editing (equal). **Wendi Zhang:** Data curation (equal); writing – review and editing (equal). **Yiran Liu:** Visualization (equal); writing – review and editing (equal). **Qiong Chen:** Software (equal); visualization (equal); writing – review and editing (equal). **Xiaobo Tao:** Software (equal); writing – review and editing (equal). **Siqi Li:** Methodology (equal); writing – review and editing (equal). **Shenxuan Zhou:** Methodology (equal); writing – review and editing (equal). **Anhui Ning:** Validation (equal); writing – review and editing (equal). **Zhenyu Li:** Visualization (equal); writing – review and editing (equal). **Tian Tian:** Supervision (equal); writing – review and editing (equal). **Lei Zhang:** Validation (equal); writing – review and editing (equal). **Jiahua Cui:** Supervision (equal); writing – review and editing (equal). **Guangyu Tian:** Validation (equal); writing – review and editing (equal). **Minjie Chu:** Funding acquisition (equal); methodology (equal); supervision (equal); validation (equal); writing – original draft (equal); writing – review and editing (equal).

## FUNDING INFORMATION

This work was supported by the National Natural Science Foundation of China (82273715, 82203771), the National Key Research and Development Program of China (2022YFC2503202), the Science and Technology Project of Nantong City (MS22022062, MS22022092, JC22022002, JC22022004), and the Graduate Research and Innovation Projects of Jiangsu Province (KYCX23‐3436).

## CONFLICT OF INTEREST STATEMENT

The authors declared no potential conflict of interest.

## ETHICS STATEMENT

The study was reviewed and approved by the ethics committee of Nantong University (Approval No. 2022‐2, February 2022).

## CONSENT

Informed consent was obtained from all subjects involved in the study.

## Supporting information


Table S1.

## Data Availability

The datasets generated during and/or analyzed during the current study are not publicly available but are available from the corresponding author on reasonable request.
