# Supplementary material for: Identification of novel pQTL‐SNPs associated with lung adenocarcinoma risk: A multi‐stage study
Source: Cancer Med. 2024 Sep 18;13(17):e70247. doi: 10.1002/cam4.70247 (PMC11409194; doi:10.1002/cam4.70247)
Supplement: Supplementary file 1 — Table S1. [file CAM4-13-e70247-s001.docx]

**Table S1. The detailed information of 80 selected pQTL-SNPs**

| No. | SNP | Gene | CHR | Position(hg37) | Minor allele | Major allele | MAF(CHB) | *P* ^a^ |
| --- | --- | --- | --- | --- | --- | --- | --- | --- |
| 1 | rs12071124 | *FH* | 1 | 241683705 | C | A | 0.41 | 0.146 |
| 2 | rs1194278 | *NEGR1* | 1 | 72714220 | C | T | 0.15 | 0.484 |
| 3 | rs226249 | *PARK7* | 1 | 8021778 | T | C | 0.31 | 0.422 |
| 4 | rs61783196 | *PRDX1* | 1 | 46291790 | C | T | 0.28 | 0.903 |
| 5 | rs11211133 | *PRDX1* | 1 | 45991079 | G | A | 0.05 | 0.802 |
| 6 | rs74418956 | *CAPG* | 2 | 85644432 | C | A | 0.09 | 0.355 |
| 7 | rs2646260 | *COL6A3* | 2 | 238277795 | A | G | 0.24 | 0.008 |
| 8 | rs2029998 | *COL6A3* | 2 | 238220307 | A | G | 0.35 | 0.333 |
| 9 | rs5030044 | *KNG1* | 3 | 186449122 | A | G | 0.22 | 0.111 |
| 10 | rs5030095 | *KNG1* | 3 | 186461349 | G | C | 0.26 | 0.517 |
| 11 | rs1656939 | *KNG1* | 3 | 186432744 | A | T | 0.43 | 0.587 |
| 12 | rs56703348 | *KNG1* | 3 | 186470523 | T | C | 0.22 | 0.782 |
| 13 | rs5030111 | *KNG1* | 3 | 186455478 | C | T | 0.08 | 0.135 |
| 14 | rs710446 | *KNG1* | 3 | 186459927 | T | C | 0.26 | 0.347 |
| 15 | rs930814 | *PROS1* | 3 | 93974928 | G | C | 0.47 | 0.306 |
| 16 | rs9290378 | *PROS1* | 3 | 93580976 | T | G | 0.46 | 0.960 |
| 17 | rs7674623 | *ANTXR2* | 4 | 80794681 | C | T | 0.06 | 0.037 |
| 18 | rs10024534 | *ANTXR2* | 4 | 80774104 | G | A | 0.19 | 0.091 |
| 19 | rs11098925 | *ANTXR2* | 4 | 80797596 | G | A | 0.41 | 0.145 |
| 20 | rs9996178 | *ANTXR2* | 4 | 80741698 | C | G | 0.10 | 0.145 |
| 21 | rs7683000 | *BST1* | 4 | 15647074 | A | G | 0.21 | 0.039 |
| 22 | rs4263397 | *BST1* | 4 | 15739390 | T | G | 0.40 | 0.585 |
| 23 | rs73224660 | *BST1* | 4 | 15714762 | G | A | 0.20 | 0.014 |
| 24 | rs6845597 | *BST1* | 4 | 15727568 | G | T | 0.49 | 0.670 |
| 25 | rs12646913 | *BST1* | 4 | 15739276 | A | G | 0.08 | 0.846 |
| 26 | rs7681694 | *SPARCL1* | 4 | 88462729 | G | A | 0.31 | 0.541 |
| 27 | rs7671511 | *SPARCL1* | 4 | 88449181 | C | T | 0.37 | 0.049 |
| 28 | rs6889109 | *HINT1* | 5 | 130474409 | T | G | 0.22 | 0.504 |
| 29 | rs2304058 | *PDGFRB* | 5 | 149508544 | C | G | 0.46 | 0.462 |
| 30 | rs2260000 | *APOM* | 6 | 31593476 | A | G | 0.19 | 0.252 |
| 31 | rs550671 | *APOM* | 6 | 31944593 | C | T | 0.41 | 0.542 |
| 32 | rs2894186 | *APOM* | 6 | 31206868 | G | C | 0.45 | 0.936 |
| 33 | rs805295 | *APOM* | 6 | 31675297 | C | T | 0.06 | 0.276 |
| 34 | rs4708089 | *CD109* | 6 | 74514153 | A | G | 0.10 | 0.850 |
| 35 | rs12215671 | *CD109* | 6 | 74435607 | T | C | 0.40 | 0.576 |
| 36 | rs1392916 | *CD109* | 6 | 74518202 | C | T | 0.41 | 0.865 |
| 37 | rs57799429 | *CD109* | 6 | 74509578 | G | C | 0.50 | 0.854 |
| 38 | rs1652492 | *PLG* | 6 | 161201024 | G | A | 0.11 | 0.166 |
| 39 | rs10945684 | *PLG* | 6 | 161110273 | C | T | 0.31 | 0.120 |
| 40 | rs4252159 | *PLG* | 6 | 161160644 | G | A | 0.33 | 0.191 |
| 41 | rs2856448 | *TNXB* | 6 | 32014575 | A | G | 0.35 | 0.791 |
| 42 | rs387608 | *TNXB* | 6 | 31941557 | G | A | 0.15 | 0.975 |
| 43 | rs1433948 | *ENPP2* | 8 | 120706244 | C | A | 0.45 | 0.097 |
| 44 | rs117980424 | *ENG* | 9 | 130554222 | C | T | 0.10 | 0.463 |
| 45 | rs11794565 | *ENG* | 9 | 130601277 | C | T | 0.41 | 0.501 |
| 46 | rs10985196 | *GSN* | 9 | 124033044 | C | A | 0.20 | 0.448 |
| 47 | rs77938199 | *ITIH2* | 10 | 7742467 | A | G | 0.22 | 0.169 |
| 48 | rs11255328 | *ITIH2* | 10 | 7782243 | A | G | 0.26 | 0.786 |
| 49 | rs2038408 | *NRP1* | 10 | 33498036 | C | T | 0.34 | 0.273 |
| 50 | rs2776937 | *NRP1* | 10 | 33606189 | A | G | 0.48 | 0.049 |
| 51 | rs2506150 | *NRP1* | 10 | 33483308 | G | A | 0.15 | 0.398 |
| 52 | rs12412765 | *PLXDC2* | 10 | 20598372 | T | A | 0.38 | 0.292 |
| 53 | rs12292693 | *CAPNS1\|CAPN1* | 11 | 64936719 | A | C | 0.08 | 0.952 |
| 54 | rs1195958 | *CAPNS1\|CAPN1* | 11 | 64936810 | C | T | 0.37 | 0.736 |
| 55 | rs2012801 | *NCAM1* | 11 | 112996454 | A | G | 0.17 | 0.494 |
| 56 | rs61902388 | *NCAM1* | 11 | 112642075 | C | T | 0.22 | 0.485 |
| 57 | rs11214489 | *NCAM1* | 11 | 112975934 | C | T | 0.48 | 0.091 |
| 58 | rs2288158 | *NCAM1* | 11 | 113133676 | G | T | 0.31 | 0.621 |
| 59 | rs2510290 | *PRCP* | 11 | 82546916 | A | T | 0.41 | 0.941 |
| 60 | rs2229437 | *PRCP* | 11 | 82564294 | T | G | 0.11 | 0.758 |
| 61 | rs12146727 | *C1S* | 12 | 7170336 | G | A | 0.08 | 0.466 |
| 62 | rs11054859 | *CD163* | 12 | 7769776 | G | A | 0.31 | 0.919 |
| 63 | rs10459147 | *LUM* | 12 | 91409672 | C | T | 0.11 | 0.788 |
| 64 | rs77751442 | *LUM* | 12 | 91485015 | G | T | 0.27 | 0.728 |
| 65 | rs7296835 | *LUM* | 12 | 91459060 | C | T | 0.12 | 0.572 |
| 66 | rs13312813 | *LUM* | 12 | 91575920 | C | G | 0.14 | 0.552 |
| 67 | rs2071065 | *TPI1* | 12 | 6977069 | T | C | 0.41 | 0.127 |
| 68 | rs2298087 | *ESD* | 13 | 47351403 | T | C | 0.20 | 0.205 |
| 69 | rs116869551 | *HAGH* | 16 | 1876619 | C | T | 0.11 | 0.794 |
| 70 | rs10083731 | *HAGH* | 16 | 1860661 | A | G | 0.10 | 0.264 |
| 71 | rs6500599 | *VASN* | 16 | 4474514 | G | C | 0.40 | 0.956 |
| 72 | rs4363 | *ACE* | 17 | 61574492 | G | A | 0.29 | 0.697 |
| 73 | rs62069916 | *APOH* | 17 | 64280503 | C | T | 0.32 | 0.047 |
| 74 | rs5112 | *APOC1* | 19 | 45430280 | C | G | 0.35 | 0.922 |
| 75 | rs5117 | *APOC1* | 19 | 45418790 | T | C | 0.23 | 0.902 |
| 76 | rs5498 | *ICAM1* | 19 | 10395683 | A | G | 0.26 | 0.456 |
| 77 | rs6036977 | *APMAP* | 20 | 24901668 | C | T | 0.46 | 0.480 |
| 78 | rs6017444 | *YWHAB* | 20 | 43528521 | G | A | 0.06 | 0.637 |
| 79 | rs136743 | *FBLN1* | 22 | 45928746 | A | C | 0.35 | 0.110 |
| 80 | rs1040402 | *FBLN1* | 22 | 45846965 | G | A | 0.12 | 0.657 |

^a^ Logistic regression analysis adjusted for age in FLCCA GWAS.

SNP, single nucleotide polymorphism; CHR, chromosome; MAF, minor allele frequency; CHB, Chinese Han population
